# Tuberculosis in the Russian Federation: Dynamics of the Epidemic Indicators before and after COVID-19 Pandemic

**DOI:** 10.3390/life12101468

**Published:** 2022-09-21

**Authors:** Anna Starshinova, Irina Dovgalyk, Mikhail Beltukov, Yulia Zinchenko, Anzhela Glushkova, Anastasia Y. Starshinova, Natalia Doktorova, Dmitry Kudlay

**Affiliations:** 1 Research Department, Almazov National Medical Research Centre, Ministry of Health of the Russian Federation, 197341 Saint-Petersburg, Russia; 2 Saint Petersburg Research Institute of Phthisiopulmonology, 191036 Saint-Petersburg, Russia; 3 Saint Petersburg State Electrotechnical Institute, 191036 Saint-Petersburg, Russia; 4 V.M. Bekhterev National Research Medical Center for Psychiatry and Neurology, 192019 Saint-Petersburg, Russia; 5 Saint Petersburg State Pediatric Medical University, 194100 Saint-Petersburg, Russia; 6 GSC “GENERIUM”, 119991 Moscow, Russia; 7 Department of Pharmacology, I.M. Sechenov First Moscow State Medical University, 119991 Moscow, Russia; 8 SSC Immunology Institute, 115552 Moscow, Russia

**Keywords:** Tuberculosis, COVID-19 pandemic, epidemiological indicators, morbidity, mortality, X-ray examination

## Abstract

The measures taken against tuberculosis (TB) in recent years in the Russian Federation have been highly effective. Unfortunately, the COVID-19 pandemic may seriously undermine the progress that has been made in the fight against TB. The aim of this study was to assess changes in the epidemiological rates of tuberculosis in the Russian Federation before and after the COVID-19 pandemic. Materials and methods. The analysis was conducted by considering the main epidemiological indicators of tuberculosis, according to the federal statistics for the period from 2017 to 2021. The parameters were estimated according to the data received from 11 areas in the North-Western region. Statistical analysis was carried out using the free software computing environment R (v.3.5.1) and the commercial software package Statistical Package for the Social Sciences (SPSS Statistics for Windows, version 24.0, IBM Corp., 2016). Research results. We found a positive correlation between the incidence among the overall population and the incidence among children aged 0–17, inclusively (r = 0.55 in 2017, r = 0.60 in 2020, and r = 0.53 in 2021). Along with the received regularities, a different trend is shown in the data analysis of general incidence and health X-ray examination for tuberculosis among the general population. The correlation has decreased threefold from 2017 (r = 0.72) to 2020 (r = 0.32); this negative trend might be the result of factors such as the quality of X-ray screening examinations among the general population, and the reduced assessment objectivity of the tuberculosis incidence rate. Conclusions. In assessing the correlation between general incidence and incidence in children under 17 years of age, as well as between incidence and mortality in the Russian Federation, a positive correlation was found with an increasing trend. Such a discrepancy might be due to decreases in the occupational health examination coverage among the general population. Therefore, in the years ahead, we can expect epidemiological indicators to increase incidence and mortality, including child mortality, associated with the insufficient detection of tuberculosis among the population during the COVID-19 pandemic.

## 1. Introduction

At the beginning of the 21st century, tuberculosis continues to be an urgent problem which is addressed at different levels. Being one of the main mortality factors in the world, tuberculosis (TB) is still a marker of a country’s social and economic wellbeing [1,2].

Today, it would be incorrect to state that the fight against the prevalence of tuberculosis is sufficiently effective or successful, despite the adaptation of different international projects and programs focused on reducing tuberculosis incidence around the world (such as the Directly Observed Treatment Short-term (DOTS) program, the Stop TB Strategy, and the Global Plan to Stop TB for 2006-2015) [3]. 

In 2014, the World Health Organization (WHO) introduced a new END TB strategy, and global goals were set up for the entire global community: to reduce the absolute number of deaths from TB by 90%, and to reduce the incidence of TB by 80% (new cases per 100,000 people per year) by 2035, which means the eradication of the disease around the world [4,5]. 

As part of this global preventative strategy for the treatment and control of TB for the period after 2015, the WHO work plan was defined as having four key areas [6]:

− The early diagnosis of tuberculosis;
− The treatment and support of patients with TB;
− The management of comorbidities of tuberculosis with Human Immunodeficiency Virus (HIV) infection;
− The prevention of and vaccination against TB.

The significance of the tasks set was emphasized at the First Global Ministerial Conference, “End of TB in the Age of Sustainable Development: A Multisectoral Approach”, which was held in Moscow in November 2017, with the participation of experts from around the world, and at the United Nations Assembly in September 2018 in New York [7].

More than 85 countries from around the world, including the Russian Federation, have signed this strategy [8]. 

The pandemic caused by the novel coronavirus in 2019 (COVID-19) had a significant impact on tuberculosis incidence and mortality [9]. This is evidenced by the results of mathematical modeling of the epidemic, which predicted TB mortality to increase by 10% and HIV-infection mortality to increase by 20%; these increases are associated with a decrease in diagnostic measures within the existing programs and treatment of the disease [10,11]. 

According to the WHO Report on Global TB Control in 2021, the COVID-19 pandemic has seriously undermined the progress that was previously made in the fight against this disease worldwide: for the first time in more than a decade, tuberculosis death rates have increased [12,13]. 

In 2020, compared to 2019, the number of tuberculosis patients who have died has increased; meanwhile, the number of tuberculosis patients receiving diagnosis, treatment, or preventive therapy has decreased significantly, and the common financial parameters of the main types of tuberculosis care have fallen [14,15,16]. 

According to the WHO forecasts, tuberculosis incidence and mortality rates will be much higher in 2021 and in 2022 [12]. The WHO estimates that about 4.1 million tuberculosis patients have not yet been diagnosed or have not been included in official national statistics. At the same time, the number of patients receiving treatment for drug-resistant tuberculosis decreased by 15%, from 177,000 people in 2019 to 150,000 people in 2020 [17]. 

Studies have shown that the number of examined and treated TB patients has decreased to 42%; the determination of new tuberculosis cases has decreased to 84%, and no persons with latent tuberculosis infection (LTBI) have been detected and observed to 95% during the last year in the 165 countries [18]. 

Since 2018, the WHO has been paying special attention to the problem of detecting and treating tuberculosis among children [8,12]. In 2020, 226,000 children under the age of 15 years died from tuberculosis. Modeling has shown that 80% of deaths from tuberculosis occur in children under 5 years of age, and 96% of children who die from tuberculosis received no treatment [19]. 

The aim of this study was to assess changes in the epidemiological rates of tuberculosis in the Russian Federation before and after the COVID-19 pandemic.

## 2. Materials and Methods

An observational epidemiological study was conducted. The analysis was based on the main epidemiological indicators of tuberculosis, according to federal statistics forms in 11 districts of the North-Western region for the period from 2017 to 2021. Annual figures are estimated per 100,000 of the average annual population, including children (from 0 to 17 years), information about which was obtained from the open demographic data of state statistics [20]. 

The parameters were estimated according to the data received upon request from 11 districts of the North-Western region (Arkhangelsk (Arkh), Vologda (Vol), Kaliningrad (Kal), Leningrad (Len), Murmansk (Mur), Novgorod (Nov), Pskov (Psk) regions and the Republics of Karelia (Kar) and Komi (Komi), the cities of St. Petersburg (St. Petersburg), and the Nenets Autonomous Okrug (NAO)).

### Statistical Analysis

Statistical analysis was carried out using the free software computing environment R (v.3.5.1) and the commercially available Statistical Package for the Social Sciences (SPSS Statistics for Windows, version 24.0, IBM Corp., 2016). The incidence rate in each individual region of the Russian Federation was calculated separately, in accordance with data from the officially published statistical materials of the Russian Federation. The degrees of association between proportions were assessed using confidence intervals, as well as the χ^2^ test with Yates’ correction. For determination of the connections between the signs, Spearman’s correlation analysis was used, and the Student’s *t*-test was calculated. Differences or association rates were considered statistically significant at a p level of less than 0.05. 

Hierarchical cluster analysis and k-means clustering were also used. The cluster analysis was carried out by taking into account the following epidemic indicators: coverage with preventive examinations of the population, general tuberculosis incidence, tuberculosis incidence among the adult population, incidence among children (0–17 years), TB mortality. 

The lowest values of the listed indicators were chosen as the criteria for epidemic wellbeing, and the region with the highest indicators after the analysis was considered to be epidemically unfavorable [21]. The presentation of the data obtained was carried out in accordance with generally accepted recommendations [22].

## 3. Results

In order to identify the correlations between the most significant epidemiological indicators, an analysis of data from the regions of the North-Western Federal District from over the last few years was carried out. 

In the first stage, we analyzed the incidence rates of tuberculosis and the incidence among children (0 to 17 years) per 100,000 people in the regions of the North-Western Federal District from 2017 to 2021 (Table 1).

**Figure 1:**
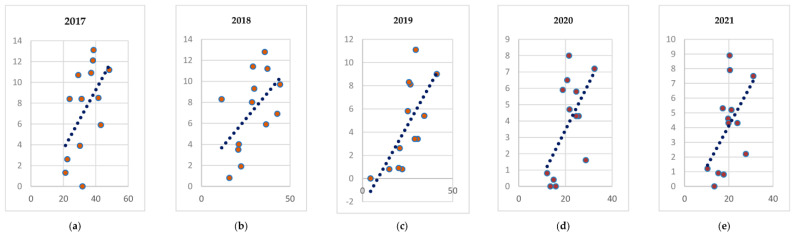
Correlation between the incidence (Y) and incidence rates among children (X) (from 0 to 17 years) per 100,000 people in the regions of the North-Western Federal District from 2017 to 2021 (**a**) 2017; (**b**) 2018; (**c**) 2019; (**d**) 2020; (**e**) 2021.

Despite a certain discrepancy between the data presented, the correlation analysis of the overall tuberculosis incidence in the population and the tuberculosis incidence among children, as shown in Figure 1, shows a positive correlation between these indicators for all years (2017–2021). 

A correlation analysis was also carried out for the incidence and mortality data for tuberculosis in the regions of the North-Western Federal District from 2017 to 2021; these data are presented in Table 2. 

**Figure 2:**
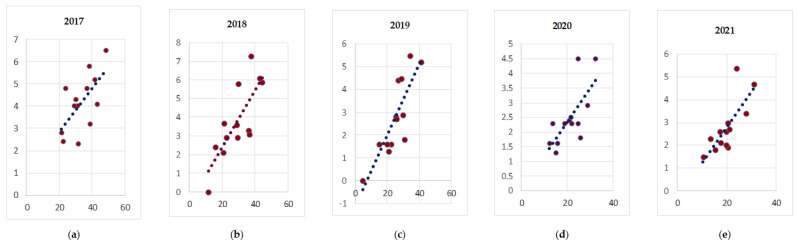
Correlation between tuberculosis incidence (Y) and mortality (X) per 100,000 people in the regions of the North-Western Federal District from 2017 to 2021 (**a**) 2017; (**b**) 2018; (**c**) 2019; (**d**) 2020; (**e**) 2021.

The following correlation analysis between the incidence and mortality rates from tuberculosis in the regions of the North-Western Federal District demonstrates the presence of a direct correlation, which is shown in Figure 2.

As shown in Figure 2, there is a clear relationship between incidence and mortality rates, with a general increase in the correlation values from 2017 to 2021 in the region. 

Table 3 presents the indicators of the correlation between the indicators. 

As shown in Table 3, we can observe a positive correlation with an increase in the correlation indicator from 2017 to 2021 in terms of the incidence among the population, and the incidence among children aged 0–17, inclusively (r = 0.55 in 2017, r = 0.60 in 2020, and r = 0.53 in 2021). 

More delineated links were noted between incidence and mortality among the population, with the correlation increasing from 0.64 (in 2017) to 0.76 (in 2021). Along with the received regularities, a different trend is shown in the data analysis of general incidence and coverage by occupational health examination for tuberculosis among all populations.

The correlation coefficient decreased threefold from 2017 (r = 0.72) to 2021 (r = 0.32); this negative trend might be a result of factors such as quality of screening examinations among the overall population, and reductions in the assessment objectivity of the TB incidence rate. 

## 4. Discussion

Until 2020, the Russian Federation was among the top 10 countries with a high burden of tuberculosis, accounting for 70% of the global burden of the disease; additionally, there was a constant increase in drug-resistant tuberculosis and HIV infection [23]. According to the WHO, the incidence of the disease in the Russian Federation in 2015 accounted for 35.6% of all cases reported in the WHO European Region, where the reduction of TB is a priority [3]. In recent years, with the support of the government, new molecular genetic methods for diagnosing *M. tuberculosis* deoxyribonucleic acid (DNA) [24], as well as bacteriological methods (BACTEC MGIT), have been introduced in the Russian Federation. Additionally, new protocols have been developed for the treatment of drug-resistant tuberculosis with the use of bedaquiline [25], linezolid, and thioureidoiminomethylpyridinium perchlorate, all of which have been approved for use [26,27]. New methods of immunodiagnostics have been introduced (such as testing with a recombinant tuberculosis allergen (Diaskintest^®^)), ELISPOT, QuantiFERON-TB Gold) [28,29], and computed tomography has been introduced into the diagnostic protocol. 

Taking into account the international situation, there was an increase in the proportion of the population receiving preventive examinations for TB; moreover, medical care programs were created to support TB patients, including those infected with HIV, which led to a decrease in the number of severe forms of TB among newly diagnosed patients, as well as a decrease in morbidity and mortality from the disease [30]. 

**Figure 3:**
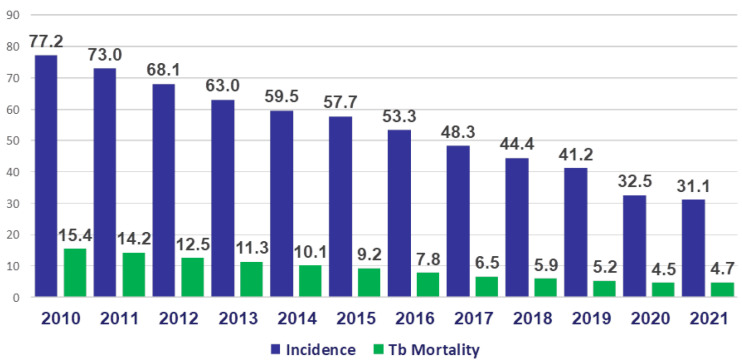
Tuberculosis incidence and morbidity per 100,000 population in the Russian Federation from 2010 to 2020.

The measures against tuberculosis taken in recent years in the Russian Federation were highly effective, and made it possible to systematically reduce the incidence rate from 77.2 to 32.4 per 100,000 population from 2017 to 2020, and the mortality rate from 15.4 to 5.1 per 100,000 population (Figure 3) [14,20,31,32]. 

This progress was also reflected in the reduction in TB incidence among children and adolescents; in 2020, according to the Federal Monitoring Center, this amounted to 6.2 and 12.7 cases per 100,000 people, respectively [14,29,32,33]. The results obtained might not objectively reflect the epidemiological situation, which may subsequently lead to the incidence rate increasing and the emergence of severe forms of tuberculosis in both the adult population and in children in the most disadvantaged regions. 

However, the COVID-19 pandemic has had an impact on TB care both worldwide [34,35]. In the Russian Federation, the COVID-19 pandemic and associated lockdown had a significant impact on the system for actively detecting tuberculosis patients; this may subsequently negatively affect the epidemic situation, leading to an increase in morbidity and the emergence of severe forms of TB in both adults and children in the most disadvantaged regions [14,32]. This trend can be identified in the results of our study in the form of a decrease in the correlation between general morbidity and preventive examinations of the population. In our study, positive correlations between incidence and mortality, as well as between general incidence and morbidity among children, may also indicate the insufficient quality of methods for the active detection of TB. This finding is consistent with the data of other authors [14,36] and with historical trends in the development of the disease [31,37].

Another important point concerns the assessment of the spread of tuberculosis in the pediatric population, which reflects tuberculosis control in the adult population [38,39]; this is shown in the obtained results, which demonstrate a positive relationship between morbidity in adults and morbidity in children. Insufficient detection of LTBI in the pediatric population is also associated with the restructuring of the health system during the pandemic, and—being a kind of reservoir of infection—poses risks for further reactivation of the process in adulthood [19,29].

An increase in TB mortality due to the COVID-19 pandemic may also be associated with a decrease in household income and a deterioration in access to medicines during lockdown, as well as a shift in healthcare spending towards the fight against COVID-19; in turn, this may lead to an increase in drug-resistant *M. tuberculosis* [40,41]. In addition to associated organizational and economic difficulties, the detection of active TB during the coronavirus pandemic was also associated with clinical features: patients with LTBI and active TB are at greater risk of infection from COVID–19 and the development of severe forms of the disease. Moreover, the differential diagnosis of these two diseases is difficult due to the similarity of the clinical and X-ray pictures [42].

It should be noted that, in the late 1980s, the coverage by occupational health examination for tuberculosis among the general population was 75%, which made it possible to achieve the most favorable incidence of TB by the end of the 1990s: 34.0 per 100,000 population [43]. In 2021, the coverage of periodic health examination for the population in the North-Western Federal District was 58.7%, which was the lowest of all the federal districts. However, against the background of this indicator decreasing both in the North-Western Federal District and in the Central Federal District, all epidemic indicators are at their lowest throughout all the federal districts in the Russian Federation [31,32]. It is expected that, with low X-ray examination coverage among the population in this district, the incidence rates will not reflect reality, due to the lack of information about pathological changes in the lungs in the unexamined population [41]. 

This analysis is based on the results of available indicators; however, the results might change due to the increase in preventive fluorography examinations among the general population, which decreased significantly against the backdrop of the COVID-19 pandemic in 2020 [13,18,34,37]. However, the epidemiological indicators from 2021 demonstrate very clearly that a slight decrease in the proportion of the population covered by preventive examinations in 2020, with a well-functioning screening system before the pandemic, did not reduce the rate of tuberculosis incidence and mortality in the Russian Federation.

## 5. Conclusions

The assessment of the epidemiological data related to tuberculosis before the start of the COVID-19 pandemic and after 2020 showed a decrease in TB incidence and mortality rates in the Russian Federation generally, and in the North-Western Federal District specifically. At the same time, according to the WHO Global Tuberculosis Report of 2021, there has been an increase in the mortality rates from tuberculosis around the world. 

However, in conducting a correlation assessment between general incidence and incidence in children under 17 years, as well as between incidence and mortality in the Russian Federation, a positive correlation was found with an increasing trend. Such a discrepancy might be due to a decrease in the proportion of the population covered by occupational health examinations. Thus, when determining the correlation coefficient between incidence and preventive coverage by occupational health examination among the population of the Russian Federation, a significant decrease was revealed from 2017 to 2021. 

Therefore, in the years ahead, we can expect epidemiological indicators of incidence and mortality to increase, including child mortality, due to the insufficient detection of TB among the population during the COVID-19 pandemic.

The obtained data can be the basis for further studies assessing the impact of various clinical and epidemiological indicators in TB. Taking into account the fact that, in the coming years, there may be an increase in the ratestable of TB and the reactivation of latent infections that were not detected in time, a more thorough investigation of risk groups and an increase in the pace of preventive examinations of the population is required; this will neutralize the harm caused in the field of tuberculosis control by the pandemic and lockdown.

The limitation of the study is that we analyzed only some of the indicators that affect the epidemiological rates of tuberculosis. The inclusion of clinical manifestations, diagnoses, and treatment outcomes, including MDR and XDR, may deepen our understanding of what really happened to tuberculosis patients during the pandemic; further studies are therefore required.

## Figures and Tables

**Table 1:** Incidence among the population and incidence among children (from 0 to 17 years) per 100,000 people in the regions of the North-Western Federal District (2017–2021).

Years/Region	2017		2018		2019		2020		2021	
	Incidence among Population	Incidence among Children (0–17 Years)	Incidence among Population	Incidence among Children (0–17 Years)	Incidence among Population	Incidence among Children (0–17 Years)	Incidence among Population	Incidence among Children (0–17 Years)	Incidence among Population	Incidence among Children (0–17 Years)
Russian Federation	48.3	11.2	44.4	9.7	41.2	9.0	32.5	7.2	31.1	7.5
North-Western Federal District	31.3	8.4	28.6	8.0	25.2	5.8	21.8	4.7	19.7	4.6

**Table 2:** Tuberculosis incidence and mortality for the population per 100,000 people in the regions of the North-Western Federal District (2017–2021).

Years/Region	2017		2018		2019		2020		2021	
	Incidence	Mortality	Incidence	Mortality	Incidence	Mortality	Incidence	Mortality	Incidence	Mortality
Russian Federation	48.3	6.5	44.4	5.9	41.2	5.2	32.5	4.5	31.1	4.7
North-Western Federal District	31.3	4.0	28.6	3.6	25.2	2.8	21.8	2.3	19.7	2.6

**Table 3:** Correlation between the studied tuberculosis epidemic indicators of the North-Western Federal District (2017–2021).

Year	First Parameter	Incidence	Incidence	Incidence	Incidence among Children (0–17 Years)
	Second Parameter	Coverage by Occupational Health Examination among Population	Incidence among Children (0–17 Years)	Tb Mortality	Tb Mortality
2017		0.72	0.55	0.64	0.67
2018		0.41	0.54	0.78	0.37
2019		0.09	0.69	0.84	0.64
2020		0.32	0.60	0.70	0.47
2021		0.40	0.53	0.76	0.32
